# General Nervous Debility, Induced by Decayed Teeth

**Published:** 1845-06

**Authors:** L. P. Brockett

**Affiliations:** Georgetown, Ky.


### ARTICLE V.

*General Nervous Debility, induced by Decayed Teeth.* By L. P. BROCKETT, M. D., of Georgetown, Ky.

ALTHOUGH not a practical dentist, I have long been convinced of the importance of an accurate and full knowledge of the principles of dental science, as well as of the diseases to which the teeth are liable, to every well informed general practitioner. But, alas! the great body of the medical profession are wofully ignorant on this subject. With a large proportion, the extent of their dental knowledge consists in a very meagre acquaintance with the anatomy of the teeth, and a general idea, that when a tooth aches, it should be "pulled;" which operation, in their hands, at least, consists in the "turning-out" of a tooth, with an antiquated and ill-constructed turnkey, to the manifest detriment, not only of the alveolar processes, but frequently of the jaw itself.

Were the members of the profession, generally, well acquainted with the structure, functions, and diseases of the teeth, they would not only be able to rescue many an unfortunate victim, from the hands of the ignorant, but daring and impudent charlatan, who, abandoning his trade, whether it be that of joiner, blacksmith, machinist or jeweller, "takes up" dentistry, as a more honorable and lucrative employment; but they would, also, derive great benefit, from this knowledge, in the light which it throws upon many of those nervous affections, which, otherwise, prove so harassing and tedious, to the general practitioner.

It is with the hope of inducing some of my medical brethren to devote more attention to this subject, that I am induced to offer the following cases for publication in your valuable journal.

CASE 1.—While prosecuting my medical studies with the late Dr. G. H. Abernethy, at Chester, Conn., in the summer of 1841, I visited Mrs. S. in company with him. She had been in very feeble health for a number of months; was pale, sallow, and emaciated; her pulse indicated great nervous irritability and debility; her tongue was coated with a whitish fur; she complained of great weakness, particularly in the back. She, as well as her friends, supposed that she was laboring under spinal disease; but after a careful examination, no tenderness could be detected on pressure, over any of the vertebræ. Her teeth were very much decayed, *yet she had never suffered from the tooth ache.* Tonics and nervines had been administered in great variety, and in very considerable quantities, but with little or no benefit.

Being, at the time, engaged in the perusal of Rush's works, his remarks on diseased teeth, as a source of constitutional nervous affections, occurred to my mind, and I suggested to Dr. A. the inquiry, whether this might not be the cause of the disease, under which Mrs. S. was laboring? The idea struck him forcibly, and on the day subsequent, he called upon the lady, and proposed that she should have some of her decayed teeth extracted. She consented, after some hesitation, and at three sittings he removed, I think, eighteen! From this period, a manifest improvement took place, and, in a few months, she had so completely recovered, that few persons would have recognized, in the beautiful and ruddy complexion, and full figure of Mrs. S. the pale, sallow skeleton of six months previous.

Case 2.—Mrs. W., of Deep river, Conn., was laboring under similar symptoms with Mrs. S., at the same time. These symptoms had commenced, if I recollect aright, with the birth of a child, nearly a year previous. She, too, had teeth which were very much decayed, but from which she experienced no sensible inconvenience. Encouraged by his success in the case of Mrs. S., Dr. Abernethy determined to try the effect of extraction in the case of Mrs. W., also. It was a long time, however, ere she could be induced to consent to the operation. Her consent being at last obtained, Dr. A. extracted, at short intervals, thirteen decayed teeth, and with the same beneficial effect as in the preceding case; she completely recovered, and has since twice become a mother, without the recurrence of her former malady.

Case 3.—In January, 1844, I was called to visit Miss H., a maiden lady of Clinton, Ct., nearly eighty years of age. She had been a remarkably robust woman, and had enjoyed sound health through life. When I first saw her she was delirious, with some fever, (of a low grade,) and very considerable debility. Her nervous system was remarkably irritable. Her disease was attributed to exposure to cold, while riding several miles, during a severe storm, the week previous. After a few days, her delirium ceased, but the nervous irritation and debility remained. After prescribing tonics and nervines for some weeks, without any apparent benefit, I noticed one day, on examining her tongue, that the fangs of the front teeth, both in the upper and lower jaw, were exposed for nearly three quarters of an inch, and encrusted with tartar, and moved readily backward and forward, upon the protrusion of the tongue. Satisfied that the nervous debility under which she labored, was owing, in part, at least, to the condition of these teeth, I immediately resolved upon their removal. This I accomplished at my next visit, and her health progressed rapidly from that date. After enjoying good health for several months, she was attacked with pleurisy, which was followed by hydrothorax, of which she died some two or three months since, as I have recently learned from her attending physician.

*Georgetown. Ky., April* 15, 1845.

